# Development of a bionic hexapod robot with adaptive gait and clearance for enhanced agricultural field scouting

**DOI:** 10.3389/frobt.2024.1426269

**Published:** 2024-09-18

**Authors:** Zhenghua Zhang, Weilong He, Fan Wu, Lina Quesada, Lirong Xiang

**Affiliations:** ^1^ Department of Biological and Agricultural Engineering, North Carolina State University, Raleigh, NC, United States; ^2^ N.C. Plant Sciences Initiative, North Carolina State University, Raleigh, NC, United States; ^3^ Department of Entomology and Plant Pathology, North Carolina State University, Raleigh, NC, United States

**Keywords:** precision agriculture, durability, energy efficiency, gait optimization, hexapod robot

## Abstract

High agility, maneuverability, and payload capacity, combined with small footprints, make legged robots well-suited for precision agriculture applications. In this study, we introduce a novel bionic hexapod robot designed for agricultural applications to address the limitations of traditional wheeled and aerial robots. The robot features a terrain-adaptive gait and adjustable clearance to ensure stability and robustness over various terrains and obstacles. Equipped with a high-precision Inertial Measurement Unit (IMU), the robot is able to monitor its attitude in real time to maintain balance. To enhance obstacle detection and self-navigation capabilities, we have designed an advanced version of the robot equipped with an optional advanced sensing system. This advanced version includes LiDAR, stereo cameras, and distance sensors to enable obstacle detection and self-navigation capabilities. We have tested the standard version of the robot under different ground conditions, including hard concrete floors, rugged grass, slopes, and uneven field with obstacles. The robot maintains good stability with pitch angle fluctuations ranging from −11.5° to 8.6° in all conditions and can walk on slopes with gradients up to 17°. These trials demonstrated the robot’s adaptability to complex field environments and validated its ability to maintain stability and efficiency. In addition, the terrain-adaptive algorithm is more energy efficient than traditional obstacle avoidance algorithms, reducing energy consumption by 14.4% for each obstacle crossed. Combined with its flexible and lightweight design, our robot shows significant potential in improving agricultural practices by increasing efficiency, lowering labor costs, and enhancing sustainability. In our future work, we will further develop the robot’s energy efficiency, durability in various environmental conditions, and compatibility with different crops and farming methods.

## 1 Introduction

The global population is projected to reach 9.75 billion by 2050. To feed such a large population, the world’s crop calorie production would have to increase to 1,406 trillion, an increase of 27% from 2023 (Sands et al., 2023). Modern agriculture faces global challenges such as climate change, soil degradation, water scarcity, and labor shortages (Sparrow and Howard, 2021). Subsequently, implementing agricultural automation and precision agriculture is a viable response to these challenges, which relies not only on the development of automation and information technology but also on using high-performance agricultural robots for continuous field data collection and precision operations.

Unlike industrial robots that work in predictable and controlled environments, agricultural robots have to deal with unstructured or semi-structured environments with tasks that are highly stochastic (De Baerdemaeker et al., 2001). Robots often need to be equipped with a range of sophisticated sensors to accommodate complex environments and improve motion accuracy. This reliance on sensors can result in increased robot costs. For example, the Spot Robot Dog (Boston Dynamics, Waltham, MA, United States) can carry 14 kg for 90 min but costs up to $200,000; the GO 1 EDU Robot Dog (Unitree, Hangzhou, Zhejiang, China) can work for 2 h with 3 kg payload, and it costs around $10,000. In recent years, advances in sensing technology have led to a gradual decrease in the cost of sensors and an increase in their durability. These advances have allowed researchers and companies to develop and build more affordable robots. For example, the MARS modular robotic system developed by the University of Georgia demonstrates its cost advantages (Xu and Li, 2022). The basic configuration of MARS costs approximately $2,300, which includes the robot’s motion system, hardware framework, and a minimal controller. Additionally, MARS can be optionally equipped with additional RTK-GNSS and RGB cameras to accurately navigate various physical obstacles, including ditches and tree branches, and perform data collection tasks.

Precision agriculture continues to evolve in the search for efficient, sustainable, and cutting-edge technological solutions. Traditional agricultural machinery often presents several disadvantages, including cumbersome transportation, soil compaction, land damage, and limitations imposed by varying field terrains. Therefore, smaller, precise, and flexible robots are becoming a trend. These robots can move flexibly through complex terrain while reducing compaction and damage to the soil. Moreover, the integration of automatic control, sensing, and computer technologies has facilitated advances in agricultural robot design and holds great promise for modern agriculture (Bawden et al., 2017). Although many robots are still in the developmental stage, they are showing promising results in agricultural tasks. Examples include 1) Spot irrigation and precision weeding (Bawden et al., 2017; Shahrooz et al., 2020), which help to reduce the consumption of water and pesticides, therefore increase the sustainability of agricultural production. 2) Pest and disease detection (Oberti et al., 2016; Mahlein, 2016), where the robot can promptly report areas where pests and diseases are found, allowing for early intervention of pests and diseases. 3) Fruit picking (Arad et al., 2020; Xiong et al., 2020), where the robot can pick fruits automatically using flexible mechanical grippers to make up for the lack of productivity due to labor shortage. Furthermore, a cluster of robots can be deployed over large areas of farmland, where individual units work collaboratively to enhance operational efficiency. In addition, robot clusters can improve the system’s robustness and reduce the chances of delays due to possible malfunctions of individual machines (Blender et al., 2016; Ball et al., 2015). This cluster-based method improves the reliability of agricultural operations, optimizing resource utilization and task distribution, leading to more consistent and effective farming outcomes.

Most previous research on agricultural robots has focused on wheeled robots and unmanned aerial vehicles (UAVs). The configuration of wheeled robots is ideal for autonomous navigation and harvesting in rows and columns of farmland, such as orchards, maize fields, and strawberry fields, where the ground is relatively flat, with fewer obstacles in the path and a relatively straightforward navigation line (Kim et al., 2021). However, for fields without rows and columns, wheeled robots are unsuitable for use due to their poor adaptation to terrain conditions and limited maneuverability. Additionally, the robot’s wheels may cause damage to plants as there is no clearance for a wheeled robot to drive through (Sparrow and Howard, 2021). UAVs are also gaining interest due to their low cost, portability, high efficiency, high throughput, and simplicity of operation (Barbedo, 2019; Xiang et al., 2020). However, aerial imaging systems have low accuracy because of long working distances. They can only capture top-view images and are unable to assess plant features that can only be observed in other viewpoints, for example, under the canopy. Furthermore, when UAVs operate outdoors, their flight stability is significantly affected by wind. This issue is exacerbated when there are substantial changes in load, making them prone to control instability. When flying at low altitudes, UAVs are likely to collide with numerous obstacles, such as tree branches, increasing operational risks. Therefore, drones may not be the best choice for tasks requiring precise control, such as picking and carrying operations.

Compared to wheeled robots and UAVs, legged robots have shown much better terrain adaptation and maneuverability performance. With more degrees of freedom (DOF) and multiple footing points, legged robots can pass through complex environments and have many applications in disaster rescue (Lee et al., 2020) and material transportation (Fu et al., 2021). Operators can adjust the robot’s center of gravity by precisely controlling the angle of each joint to adapt to different ground conditions. In addition, by precisely controlling the landing position of each leg, the robot can effectively avoid obstacles, thereby increasing its operational efficiency and safety in unpredictable environments. This advanced mobility and adaptability allow legged robots to play a crucial role in outdoor operations.

In the field of legged robots, quadruped robots have fewer support points compared to hexapod robots. Consequently, quadrupeds must alternately lift each leg during movement. If a suitable support point cannot be found within the robot’s accessible space, its ability to progress is limited. Additionally, the dynamic characteristics of quadrupedal motion require constant body balance maintenance, increasing the computational complexity of control algorithms and power consumption. This requirement is reflected in shorter operational endurance times. We tested a commercially available quadrupedal robot (Unitree, Hangzhou, Zhejiang, China) carrying a 3 kg payload and traveling at 2 m/s on flat concrete ground, resulting in an endurance of only approximately 30 min. In contrast, with more support points, hexapod robots provide more stable support and higher dexterity, making them more suitable for agricultural applications where higher load capacities and longer endurance are required.

Researchers have been working on robot locomotion over the past three decades (Bhatti et al., 2015). Significant advancements have been achieved in the stability and sensitivity of robot locomotion. Intensive research and experimentation have led to the development of advanced algorithms that can be dynamically adapted to speed changes and different terrain types (Raibert et al., 2008; Bretl and Lall, 2008). Numerous studies have validated the performance of hexapod robots in terms of stability, accuracy, and flexibility across various environments, including flat hard surfaces and terrains with regular obstacles (Coelho et al., 2021; Saranli et al., 2001). These investigations demonstrate that hexapod robots exhibit superior performance compared to other robotic configurations like quadruped robots. Such attributes render hexapod robots exceptionally well-suited for applications necessitating high precision and complexity. In terms of control algorithms for hexapod robots, some researchers have developed control methods based on neuromodulation mechanisms inspired by biological systems (Polykretis et al., 2020) as well as neural networks that mimic the stimulus-response of the nervous system (Lopez-Osorio et al., 2022). Meanwhile, other researchers have worked on developing machine learning-based control algorithms (Ehrlich and Tsur, 2021; Thor and Manoonpong, 2021; Jorge et al., 2020). These algorithms provide sophisticated solutions to maintain robot stability in environments with dense obstacles and uneven terrain. However, improving the stability and efficiency of legged robots in unstructured environments, such as agriculture, remains a challenge. To address this issue, a research group at Kyoto University developed a tracking robot based on ambient CO2 concentration (Iida et al., 2008). These advances provide solutions for modifying robot behavior based on external environmental inputs. In addition, researchers at ETH Zurich developed another robot that integrates stereo vision for navigation and proprioceptive terrain sensing for adaptive control (Bjelonic et al., 2018). Despite these technological advances, the widespread use of hexapod robots in agriculture still faces significant challenges, such as adapting to different soil properties, avoiding plant damage, and improving endurance, which led to the limited use of hexapod robots in agricultural practices.

Our research introduces a bionic hexapod robot with innovative control mechanisms to address the challenges of applying legged robots in agricultural scenarios. The objectives of this study are to: 1) conceptualize and create a design framework for a terrain-adaptive agricultural robot that meets predefined requirements, focusing on adaptability to different agricultural scenarios; 2) build the robot according to the established design concepts to ensure structural integrity and operational feasibility; 3) design algorithms to enable the robot to perform basic autonomous and intelligent tasks, including but not limited to obstacle avoidance and automatic gait transformation, enhancing its functionality in dynamic environments; and 4) evaluate the robot’s operational efficiency in agricultural environments and gather empirical data and insights to inform advances in more complex functions such as autonomous navigation and robot clustering strategies.

## 2 System overview

The robotic system designed in this study is based on bionic principles, which offer superior dexterity, robust load capacity, and the ability to adapt to complex agricultural terrains. The system’s overall architecture integrates three core subsystems: a sensing system (Section 3.3), a control system (Section 3.4), and a motion system (Section 3.5). Each has been specifically designed to improve the efficiency and stability of the robot in agricultural environments. The specifications are given in Table 1.

**TABLE 1:** Specifications of the robot.

Type	Description
Mass	4.2 kg (without battery), 4.7 kg (with 2 batteries)
Dimension	Diameter:1.21 m (expanded), 0.77 m (standing), Clearance: 6–18 cm
Power supply	Two 3S LiPo battery (11.1 V, 5200 mA h)
Traveling speed	Up to 1.2 m/s
Payload	8 kg (including batteries and sensors)
Endurance (with 2 batteries)	3 h (standing), 1 h (traveling at 0.5 m/s )

We offer a range of optional sensing system configurations to accommodate varying mission requirements and enhance the robot’s capabilities. For scenarios necessitating an assessment of the robot’s kinematic performance, we equip the standard version of the robot with IMU and force sensors to record comprehensive data on the robot’s attitude and motion dynamics. Accordingly, we have developed an advanced version of the robot for scenarios that require autonomous navigation, with an additional environmental sensing module. This module integrates multiple sensors, including LIDAR, distance sensors, and cameras, to acquire real-time data on ground conditions and potential obstacles. Such data is pivotal for enabling autonomous navigation and obstacle avoidance, thereby facilitating the robot’s efficient operation across diverse agricultural environments. In the control system, the signals from the sensors are analyzed by the data processor to generate the robot’s motion parameters such as the angle each joint needs to rotate, the robot’s motion speed, and the direction of motion according to predefined algorithms. The motion controller then generates Pulse-Width Modulation (PWM) signals recognizable by the motion system based on these parameters according to a written algorithm. The function of the motion system is to precisely regulate the angle, rotational speed, and torque of the servos according to the commands given by the control system. This regulation ensures that the robot moves at a predetermined trajectory and speed. With these three systems integrated, the robot can overcome the challenges encountered in complex agricultural scenarios.

To accommodate obstacles of different heights in the field, a notable feature of this robotic system is its ability to switch motion modes between high clearance and low clearance. This feature enhances its adaptability for plants at different growth stages and enables it to move efficiently by changing its clearance to cross obstacles of varying heights. The robot can operate in various environmental conditions, from flat terrain to sloping terrain and terrain with various obstacles.

## 3 Hardware design

### 3.1 Hardware design requirements

Since the application scenario of our robotic system is agricultural tasks, parameters such as weight, endurance, traveling speed, and payload of the robot need to be considered. Therefore, the following performance requirements are used to guide the hardware design (Xu and Li, 2022; Biswal and Mohanty, 2021; Raj and Kos, 2022).

1. Lightweight. Agricultural robots are characterized by lightweight and flexible movement compared to large machines such as tractors. Currently, most agricultural robots weigh between 5 and 800 kg. For legged robots, due to their simple structure and small size, their weight is on the small side, between 1 and 20 kg. Therefore, our goal is to ensure that the maximum weight should not exceed 10 kg while maintaining the strength and stability of the robot.
2. Endurance. The endurance time of a robot is mainly determined by the battery capacity and the power of the robot’s motion and control system. Existing legged robots have an endurance from 2 to 12 h. Therefore, the minimum endurance should be no less than 2 h.
3. Traveling speed. Agricultural robots do not need to travel at high speeds because the task requires them to be highly accurate. Most current agricultural robots travel at a speed between 0.5 and 3 m/s, and our proposed design has a maximum speed range from 0.8 to 1.5 m/s.
4. Payload. The robot needs to have enough payload to carry the necessary hardware modules. Although the payload depends on the different mission requirements, the robot we proposed at this stage should be able to carry at least equipment such as sensors, cameras, and LiDAR. Thus, the load should not be less than 8 kg.

### 3.2 Body design

**FIGURE 1:**
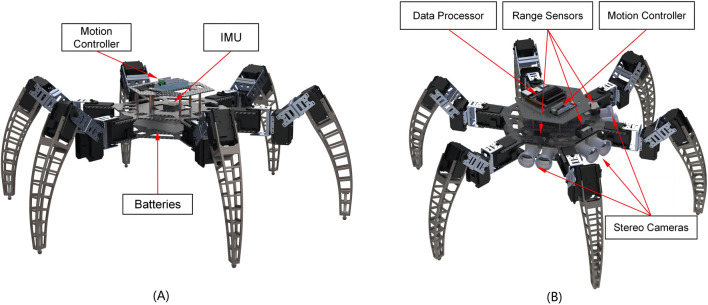
Robot CAD model. **(A)** The standard version of the robot. It has an IMU and force sensors for recording robot attitude and motion information. **(B)** The advanced version with additional hardware includes stereo cameras, distance sensors, and the data processor. The stereo cameras are used to generate depth images and are paired with distance sensors mounted on the second base plate.

**FIGURE 2:**
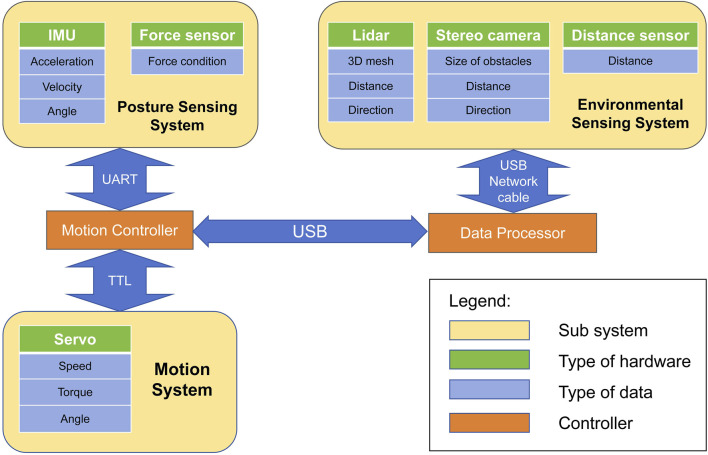
The architecture of the integrated robotic sensing and motion system. The system contains a posture-sensing system, an environmental sensing system, a motion system, and two controllers.

Inspired by the versatile locomotion capabilities of insects in unstructured environments, the robot features six highly flexible legs, each with three degrees of freedom. This design allows the robot to mimic the complex movements of natural creatures. Additionally, the robot’s architecture is modular, allowing for the flexible integration of various sensors based on specific operational requirements. This adaptability enhances the robot’s utility in different tasks and environments. Figure 1A is a CAD model of the standard version of the robot, depicting the structure of the necessary components of the robot and showing the arrangement of the bionic legs and core components. Figure 1B shows the design of the advanced version of the robot, including range sensors, stereo cameras, and a processor with higher computational power. Figure 2 shows the structure of the hardware system. The following subsections delve into the hardware design requirements and provide detailed descriptions of the design and function of each robot component.

The frame of the robot body (Figure 1 in the Supplementary Material) is used to support the robot’s overall structure and connect to other systems. The frame is rigid, and two significant criteria were considered in its design. The first criterion is the frame’s ability to withstand the robot’s total weight, and the stresses generated by the robot in motion. The second criterion is that the frame should be lightweight, and the weight of the frame should be minimized while maintaining stability to increase the load capacity of the robot. Based on the above design concept, we chose carbon fiber, known to have excellent strength-to-weight ratio and rigidity, as the material for the base plate of the robot body. The design also features a double-layer construction with six nylon struts in the center for support. This design increases the available space within the fuselage and provides enhanced resistance to bending moments. The frame is divided into three layers by a double-layered base plate, allowing the user to configure the frame with the mission’s requisite sensing and control systems. After experimental trials, the thickness of the base plate was selected to be 2 mm to ensure high stiffness and low weight.

### 3.3 Sensing system design

We developed a posture sensing module for both the standard and advanced version of the robot for real-time monitoring of the robot’s posture and motion status during movements. This configuration enables the robot to dynamically adjust its motion strategy based on the posture data and maintain balance by continuously evaluating its own posture. For the advanced version of the robot, we have introduced an additional environmental sensing system for robot perception. This system enables the robot to detect variations in the surrounding terrain and autonomously plan an appropriate path of travel, thus enabling the robot to perform advanced tasks such as autonomous navigation and obstacle avoidance.

#### 3.3.1 Posture sensing system

The posture sensing system comprises a nine-axis IMU (LORD, Cary, NC, United States) and six force sensors mounted on each leg. The IMU measures the robot’s inclination, angular velocity, and angular acceleration in pitch, yaw, and roll directions, which are used to provide feedback on the current robot posture. The force sensors are mounted at the end of each leg, and the robot’s gait algorithm stops when the force sensors detect that the leg has touched the ground. The force sensor changes its resistance under external pressure, and the controller reads the voltage of the sensor to determine whether the robot’s foot touches the ground or not. This design addresses the situation where part of the leg hovers above a groove when the robot crosses it, preventing potential falls.

#### 3.3.2 Environmental sensing system

The environmental sensing system is realized by interacting with a LiDAR (LIVOX, Shenzhen, Guangzhou, China), three Red-Green-Blue (RGB) cameras (FLIR, Wilsonville, OR, United States), and three range sensors (Benewake, Beijing, China). LiDAR provides detailed spatial data and precise ranging information with high accuracy and long-range detection capability. However, the large amount of data generated by LiDAR requires high computing power and is weather-sensitive. In this case, 3D cameras are complementary and can provide better depth perception of the scene with faster computation. In addition, the distance sensor, with its low cost, small size, and high efficiency, can provide instant distance measurement, which is especially effective for obstacle avoidance and essential object detection. This multi-sensor fusion approach optimizes the sensing system’s overall performance, providing more robust solutions in various environments and application scenarios.

The effective detection range of the LiDAR is a cylindrical space with a diameter of 20 m, with the installation height as the upper surface, excluding a cone with a top angle of 32°. The bottom surface of this cone does not exceed the projection of the base plate on the ground, so the LiDAR’s detection range is extensive enough for the robot. The camera used in this study features a diagonal field of view (FoV) of 94°, a horizontal FoV of 82.9°, and a vertical FoV of 66.5°. The base plate of the robot’s standard version was designed with three sets of camera mounting slots, each vertically offset downward by 20°. Additionally, the angle between each set is 60°. This arrangement enables the integration of the three camera sets to create a semi-panoramic image of the robot’s surroundings. This composite imaging capability facilitates the precise identification and labeling of obstacles and distinctive terrain features, enhancing the robot’s operational effectiveness in diverse environments. The range sensor has a measurement range of 20–200 cm and is used in conjunction with the LiDAR and cameras for obstacle detection.

### 3.4 Control system design

The control system consists of a motion controller and a data processor. The motion controller is the OpenRB-150 (ROBOTIS, Lake Forest, CA, United States), which supports programming with the Arduino IDE. Since the robot needs to process images and point cloud information generated by the camera and LiDAR, the amount of data is quite large and requires a processor with high computing power. Therefore, we have chosen the Jetson nano (NVIDIA, Santa Clara, CA, United States), which is lightweight and has high computational power for applications that require real-time processing of complex algorithms in the field.

### 3.5 Motion system design

**FIGURE 3:**
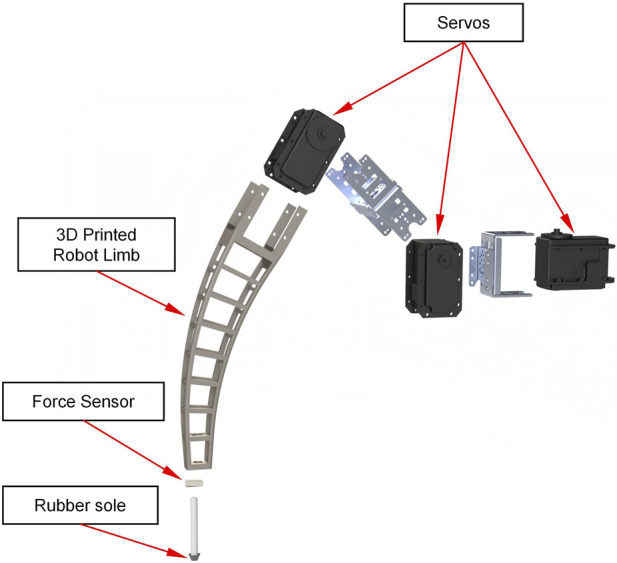
The exploded view of the whole leg. The robot’s legs contain three servos, a 3D-printed leg skeleton, pressure sensors, and rubber pads to increase friction.

The design idea of the motion system is to form a leg with three servos and three connectors. The servo in the hip joint has horizontal rotational degrees of freedom to control the forward and backward motion of the leg. The knee and ankle joints are equipped with servos with vertical rotational freedom to control the up-and-down motion of the leg. Since the design goal of the robot is to cross potential obstacles in agricultural fields, we designed the leg as a support bar with a 27 cm long support rod with a 30-degree curvature to cross obstacles more easily. The servos used are Dynamixel MX-106T (ROBOTIS, Lake Forest, CA, United States), which has a rated voltage of 12 V and a rated torque of 8.4 N**·**m. This high-performance servo provides powerful torque to increase the robot’s payload. The connecting parts that require high strength are made of aluminum alloys using CNC machining methods, and the non-stressed parts are manufactured using 3D printing technology. An exploded view of the entire leg is shown in Figure 3.

## 4 Locomotion model

In this study, we developed a Terrain-Adaptive gait (TA gait) based on the traditional tripod gait (Duan et al., 2009) to optimize the robot trajectory. Previously commonly used robot motion control is usually optimized with a fixed clearance (Coelho et al., 2021). In agricultural applications, this gait is not well adapted because of the inconsistent heights of obstacles. In contrast, our proposed terrain-adaptive gait enables the robot to vary its clearance to cross the barriers more efficiently and with less energy. Thus, it is more suitable for applications in agricultural scenarios.

### 4.1 Terrain-Adaptive gait

**FIGURE 4:**
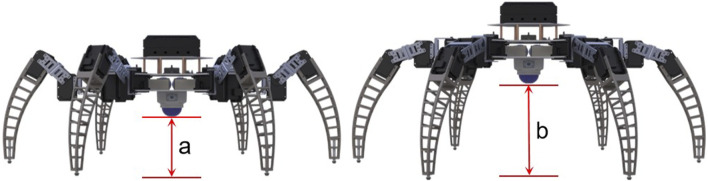
Comparison of the clearance when the robot is in marching and step-over modes. The a and b in the figure represent the clearance of the robot in these two modes, which are 6 and 18 cm, respectively.

**FIGURE 5:**
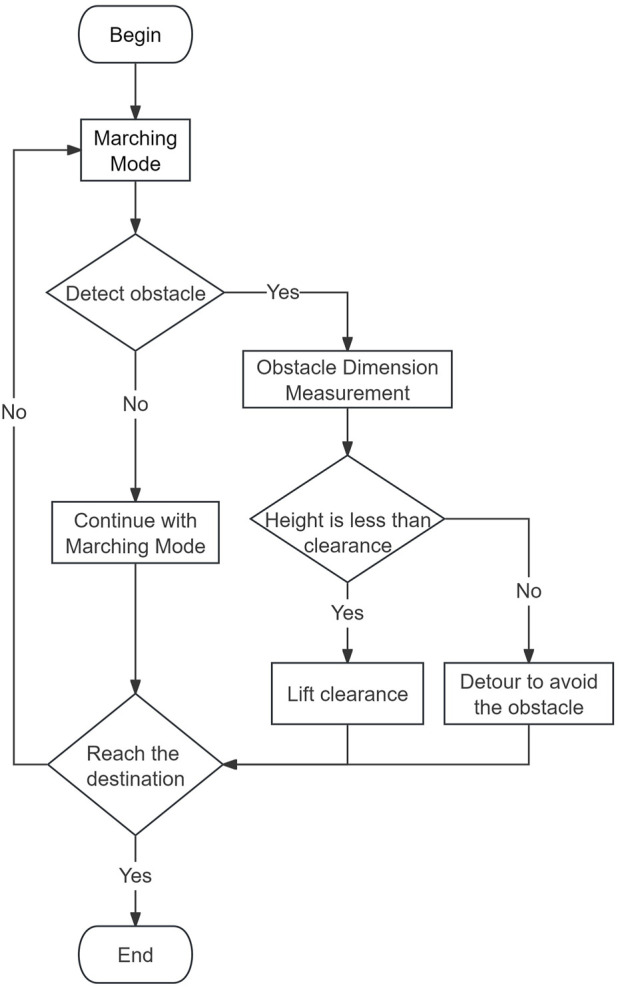
The flowchart of robot switching motion modes.

The TA gait is categorized into two modes. The first mode is a marching mode with lower clearance and lower energy consumption (Figure 4A), while the second mode is a step-over mode with higher clearance (Figure 4B). Both modes combine three basic motions of the robot’s single leg, and these motions include striding motion, dragging motion, and clearance change motion. Striding motion is the process of lifting the leg from a decent position, moving it to the target position and then stepping back to the ground. Dragging motion is defined as the process in which the leg stays in contact with the ground and pulls the robot’s body forward by friction. As the robot moves, its six legs repeat the striding and dragging motions at intervals to ensure that at least three legs are in contact with the ground at any given moment and that the center of gravity of the robot falls within the area formed by the contact points. The logic flow of the robot in accomplishing the task is shown in Figure 5.

**FIGURE 6:**
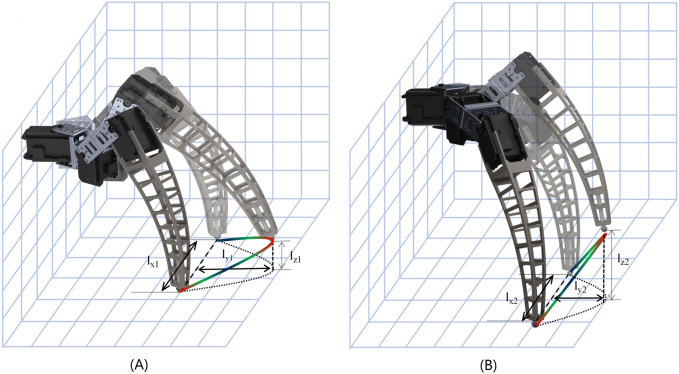
3D plot of the foot’s trajectory. These figures show the trajectory of the end of the leg as it takes a step, where l_x1_, l_x2_ is the step length, l_y1_, l_y2_ is the furthest distance outward and l_z1_, l_z2_ is the height at which the leg is raised. **(A)**. The trajectory of the end of the leg in marching mode. **(B)**. The trajectory of the end of the leg in step-over mode.

The marching mode of this robot consists of a leg-spanning motion and a dragging motion. During the spanning phase, the robot’s foot is lifted and moves along an arc on the sagittal plane, transitioning from the rear pole position to the front pole position. On the other hand, the drag motion involves the robot’s body being propelled forward through friction while the foot maintains contact with the ground at the front pole position. The robot advances one step forward by completing two sets of spanning and dragging motion cycles. The leg movement process and foot trajectory 3D plot for the marching mode and step-over mode are shown in Figures 6A, B.

The step-over mode of the robot is similar to the marching mode, but the knee servo has an angle that is greater than 90° and a larger clearance. The ankle joints are contracted inward during the movement, so more considerable obstacles can be crossed.

In Figures 6A, B, l_x1_, l_x2_ represent the forward distances, l_y1_ and l_y2_ denote the lateral extension distances of the foot, and l_z1_ and l_z2_ indicate the height of the leg lift. In the marching mode, each leg is rotated at an angle of 30°, and the height of the leg lifted is a fixed value of about 3 cm, so that the foot trajectory projected on the ground is a minor arc with an angle of 30° and a radius of the length of the leg contracted up.

In the step-over mode, the angle of rotation of each leg and the height of lifting depends on the size of the obstacle, and the value of each parameter can be inverted by the foot trajectory. In our robot, the trajectory is set as a minor arc that precisely envelopes the obstacle.

### 4.2 Leg trajectory control model

In this paper, leg trajectory control is optimized based on the traditional tripod gait algorithm. Traditional trajectory control, such as pendulum trajectory control (Yin et al., 2023), requires computing the landing point of the robot’s foot end at each step and using inverse kinematics to control the angle of each servo. This approach generates many complex computations, requires long computation time and energy consumption, and is not conducive to the longer endurance required in agricultural scenarios. Furthermore, the end of the foot makes a greater angle contact with the ground, resulting in a more profound impact. Therefore, we propose a computationally efficient trajectory control algorithm. Previous studies have explored similar curve-fitting and periodic oscillation control methods and validated their effectiveness in achieving adaptive motion (Coelho et al., 2021; Saranli et al., 2001; Iida et al., 2008). Building on these fundamental studies, our approach introduces gait switching and adjustable robot spacing, addressing the problematic leg of previous gaits that cannot adaptively cope with obstacles of different heights.

The following Equations 1–3 define the trajectory control of the robot leg:

$$\theta_{i1}=\theta_{i1}^{0}+l_{1}\sin\;\left(2t\right)$$ (1)

$$\theta_{i2}=\left\{\begin{array}{ll} \theta_{i2}^{0}+\frac{1}{2}l_{2}\left[\cos\;\left(4t\right)+1\;\right]\; & if\;\cos\;\left(2t\right)>0\\ \theta_{i2}^{0}\; & otherwise\; \end{array}\right.$$ (2)

$$\theta_{i3}=\left\{\begin{array}{ll} \theta_{i3}^{0}+\frac{1}{2}l_{3}\left[\cos\;\left(4t\right)+1\;\right]\; & if\;\cos\;\left(2t\right)>0\\ \theta_{i3}^{0}\; & otherwise\; \end{array}\right.$$ (3)

where $\theta_{i1}$ is the angle of the hip servo, $\theta_{i2}$ is the angle of the knee servo, and $\theta_{i3}$ is the angle of the ankle servo. The letter i identifies which leg of the robot this is. Symbols with a superscript of 0 represent the angle of each joint at initialization (moment t = 0). The symbols l_1_, l_2_, and l_3_ indicate the angle of the hip, knee, and ankle joints need to be rotated. These parameters can be solved by the kinematics mentioned in Section 4.1. Specifically, they can be solved by forward kinematics (Equation 4) and inverse kinematics (Equation 5):

$$\left[\begin{array}{c} x\\ y\\ z \end{array}\right]=\left[\begin{array}{ccc} \sin l_{1} & \cos l_{2}\sin l_{1} & \cos l_{3}\sin l_{1}\\ \cos l_{1} & \cos l_{2}\cos l_{1} & \cos l_{3}\cos l_{1}\\ 0 & \sin l_{2} & -\sin l_{3} \end{array}\right]\left[\begin{array}{c} a\\ b\\ c \end{array}\right]$$ (4)

$$\left\{\begin{array}{c} l_{1}\approx \sin^{-1}\left(\frac{x}{\sqrt{x^{2}+y^{2}}}\right)\\ l_{2}\approx \sin^{-1}\left(\frac{z+c\sin l_{3}}{b}\right)\\ l_{3}\approx \sin^{-1}\left(\frac{b\sin l_{2}-z}{c}\right) \end{array}\right.$$ (5)

where x , y , z represents the position of the foot landing point in the forward direction, horizontal direction and vertical direction respectively. Letter a , b , c indicate the distances between the hip and knee joints, the knee and ankle joints, and the ankle and the foot respectively.

The value $\cos\left(4t\right)$ denotes the frequency of joint rotation, embodied in the period of the robot motion, which is the velocity. The value $\cos\left(2t\right)$ determines the rotation mode of the robot’s knee joint. The equations consider the joints’ initial angle, rotational speed, and the time-dependent sinusoidal modulation factor. When $\cos\left(2t\right)$ is positive, it indicates that the leg is still in the forward-spanning phase. Otherwise, it indicates that the foot has moved to the anterior and posterior poles and should be in the dragging mode. Various motion modes can be achieved by adjusting the algorithm variables. To achieve stable movement in a straight line of the robot, the rotational directions of adjacent hip joints are set to alternate in opposite directions, and the rotation of the knee joints has a phase difference of half a cycle to maintain dynamic balance. The robot can also be oriented by rotating the hip joints in the same direction. When a passable obstacle is detected, the robot automatically adjusts the height of the base to ensure effective passage. During clearance elevation, the leg servos follow the following dynamics Equations 6–8:

$$\theta_{i1}=\theta_{i1}^{0}$$ (6)

$$\theta_{i2}=\theta_{i2}^{0}+\frac{∆y}{T}\sin\left[\frac{1}{2T}\left(t-t_{0}\right)\right]$$ (7)

$$\theta_{i3}=\theta_{i3}^{0}+\frac{∆z}{T}\sin\left[\frac{1}{2T}\left(t-t_{0}\right)\right]$$ (8)

where Δy and Δz refer to the angle of the knee and ankle joints that need to be rotated, T refers to the period of the robot traveling further, and t_0_ refers to the moment at which it starts lifting the ground.

The algorithm’s complexity can be significantly reduced by using the sinusoidal modulation factor and time as inputs to the motion function. Compared to traditional robot motion algorithms such as tripod gait, this approach eliminates the need for inverse kinematics to compute the position of the leg, thus allowing faster and more efficient computations.

## 5 Experiments and results

The TA gait allows the robot to adaptively adjust its base height, enabling it to directly cross obstacles by lifting the base instead of navigating around them. Consequently, in complex environments, robots employing the TA gait complete tasks more quickly and efficiently than those using traditional algorithms. To verify this, we conducted simulations and field tests to assess the stability of the robot’s motion in various terrain types. Additionally, field tests were carried out to evaluate energy consumption.

### 5.1 Performance criteria

The dimensionless cost of transportation (CoT) is a widely used metric for evaluating the energy efficiency of ground robots (Bjelonic et al., 2018). It is defined as following Equations 9, 10:

$$\mathrm{CoT}=\frac{UI}{mgv}$$ (9)

$$\bar{\mathrm{CoT}}=\frac{\frac{1}{T}\sum_{i=1}^{n}U_{i}I_{i}}{mg\frac{\Delta x}{\Delta t}}$$ (10)

where U is the battery’s voltage, I is the instantaneous current output from the battery, m is the mass, v is the robot’s speed, and Δt is the time required to move the distance Δx . The total power consumption calculated by CoT based on the voltage and current of the power supply includes the power of the motors, the power of the data processor, the power of the sensors, and other losses such as friction. It can be seen that energy efficiency is highly dependent on the characteristics of these power-using devices. Since this work calculates the CoT of the same robot in different environments, it is assumed that these power-using devices remain constant during the experiment.

Pitch angle and displacements, which measure the inclination of the robot’s body along its longitudinal axis, are used to evaluate the robot’s dynamic stability during operations (Chen et al., 2022). These metrics are crucial for assessing the robot’s ability to maintain balance and perform effectively across varying terrains.

### 5.2 Simulation test

We conducted simulation experiments in Simulink to test the robot’s performance on various terrains, including flat terrain with obstacles, sloping terrain, and complex terrain with multiple obstacles and slopes.

#### 5.2.1 Simulation model configuration

The robot simulation model consists of six parts: World Setting, Robot Components, Contact Force, Terrain, Parameter Monitor, and Locomotion Algorithm. The first five parts belong to the physical modeling part, which will be introduced in this section. The Locomotion Algorithm has been introduced in Section 4. The World Setting part establishes the world coordinate system for the robot and defines the ground model. The position of the robot model with respect to the ground is also set here. In the Robot Components section, the robot’s six legs are connected to the body by rigid joints, and the dynamics of the three joints of each leg are defined. Contact force contains the force between the robot and the ground and the collision between the robot’s own parts. Terrain sections define the position and size of the obstacles in the environment. The parameter monitor can display curves for each parameter of robot dynamics and kinematics over time. Details of the entire simulation environment and each module can be found in the Supplementary Material (Figures 2–6).

To build the robot simulation model, a 3D model (Figure 2) of the robot designed in SolidWorks was used and imported into Simulink through the Simscape Multibody interface. This process enables the transition from static 3D design to dynamic simulation, allowing detailed analysis and refinement of the robot’s kinematic dynamics and interaction capabilities in a controlled virtual environment. This approach improves the accuracy of simulation results and provides a suitable framework for iterative testing and optimization of robotic systems.

The world framework block, mechanism configuration block, and solution configuration block are the three most critical parts of the world setting part. The world frame block was used to define the world origin and coordinate system in the simulation environment. The mechanism configuration block was used to define the physical parameters in the simulation environment, such as gravity was initially set to 9.8 m/s^2^ in the *z*-direction, and the solver configuration block was used to control the physical simulation environment. These three components build the complete physical simulation environment. The first component connected to the World setting part is a brick solid block with dimensions of 20 m × 20 m × 0.01 m, which serves as the ground in the simulation environment. To ensure that the robot can make translational and rotational motions in three different dimensions on the ground, a 6-DOF joint block is connected between the robot model and the ground model. The distance between the robot’s coordinate system and the ground was then set using the coordinate system transformation module. The distance between the center of our robot and the ground is 0.25 m.

In the Robot Components section, the relative positions of the robot’s body and each leg and the relative positions of each component of the leg are determined from the SolidWorks model of the robot, and the Simscape Multibody plug-in automatically generates the parameters. The simulation model of the robot leg, which consists of three rotating joints and a number of rigid connectors. The three rotational joints allow the leg to rotate in the horizontal plane, around the knee joint in the vertical plane, and the ankle joint in the vertical plane, respectively. In this paper, to control the rotation of the joints, it is necessary to set the actuation properties in the revolute joint block, in which the motion is being set to be provided from the output generated by the Locomotion Algorithm and the torque is being calculated automatically.

The subsequent phase of the simulation model configuration entails the integration of the contact force module, which comprises solely spatial force blocks. This module is essential for the interaction between the robot and its environment. In particular, the spatial force block is connected to the leg model at one end and to the ground model at the other end. This block enables the generation of interaction forces between predefined objects and planes, thus ensuring that the robot model remains stable and does not penetrate the ground in simulation.

Finally, a terrain and parameter monitoring module is incorporated into the model. This facilitates modifying the terrain environment and the real-time recording of pertinent data. The terrain configuration comprises the ground, the objects, and the rigid transformations. Collectively, they define the physical properties of the terrain. The position and angle of objects relative to the center of the ground can be adjusted within the rigid transform. Furthermore, the parameter monitor is designed to track and display dynamic changes in various parameters during the simulation. This is accomplished through parameter outputs, unit conversions, and displays that collectively present the temporal evolution of parameter values.

In summary, in conjunction with the motion algorithms described in Section 4, these elements constitute a comprehensive simulation model of the robot. The model contributes to a detailed understanding of the robot’s interaction with its environment and provides a robust framework for further experimental validation and refinement.

#### 5.2.2 Moving on sloping terrain

**FIGURE 7:**
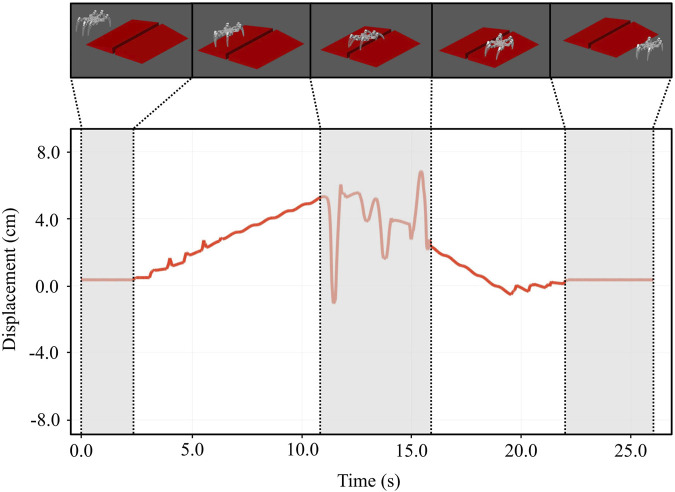
Simulation of a robot traveling uphill and downhill on a 15° inclined surface. The robot is capable of traveling steadily through slopes and can extricate itself from a grove with one leg stuck.

In the simulation environment, we introduced slopes with a gradient of 15°, including uphill, downhill, and a trench, into the terrain portion of the simulation environment. A snapshot of the simulation outcome of the robot navigating these slopes is presented in Figure 7. The figure illustrates the vertical displacement of the robot’s body as it ascends and descends the slope. It is evident that the robot’s vertical displacement remains stable, exhibiting minimal variation along the slope. Moreover, when encountering the trench, despite one leg becoming obstructed and the body being inclined, the robot successfully gets out of the trench and reverts to its original motion trajectory.

**FIGURE 8:**
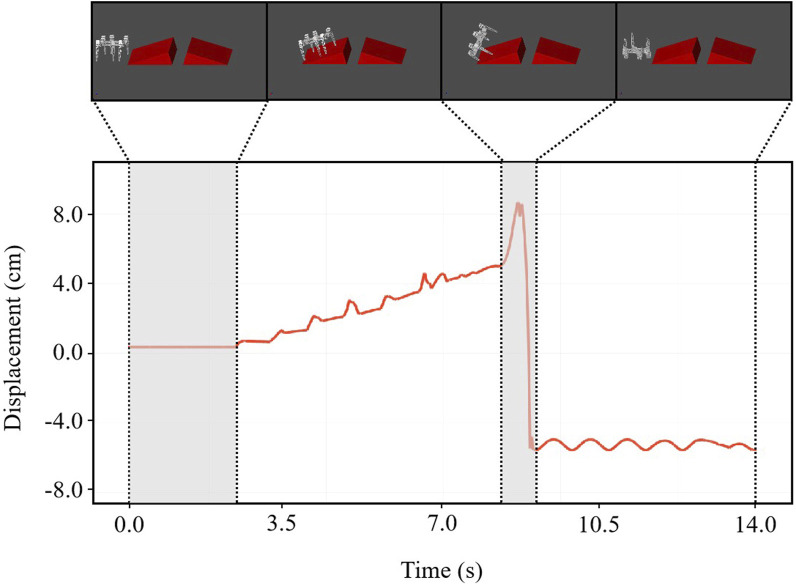
The maximum slope the robot system can travel. When the slope increases to 17°, the robot gets an unstable center of gravity and flips over.

Our simulation experiments also included evaluating the robot’s stability on various inclined surfaces to determine the maximum slope it could go over. The experimental result shows that at an angle of 17°, the robot encountered stability problems due to its center of gravity moving outside of the tripod stability zone delineated by the contact points. This situation would result in the robot tipping over. Figure 8 illustrates the fluctuation in the vertical displacement of the robot’s body during the simulation.

#### 5.2.3 Moving on terrain with multiple obstacles and slopes

**FIGURE 9:**
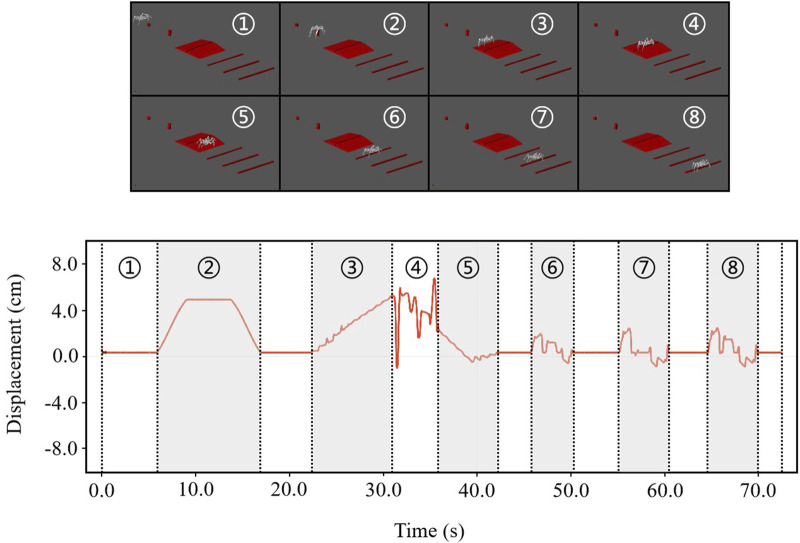
Simulation results demonstrate the legged robot’s ability to cross obstacles, navigate furrows, and climb bars. Top: Sequential simulation snapshots showcasing a legged robot navigating through a challenging obstacle course with varying terrains and obstructions. Bottom: Graphical representation of the robot’s vertical displacement over time, indicating its stability and agility during the obstacle course navigation.

We added three additional bars with cross-sectional dimensions of 6 cm by 6 cm and 80 cm spacing between the bars to the slope simulation environment in the previous section. A snapshot of the simulation results of the robot passing through the entire terrain is shown in Figure 9, which shows the vertical displacement of the robot as it passes through the different terrains.

### 5.3 Field test

We conducted numerous experiments to evaluate the performance of the previously proposed motion algorithm in terms of energy efficiency and body stability. Videos related to robot testing can be found in the Supplementary Material.

#### 5.3.1 Experimental setup

To generate experimental results in different environments, we prepared four different experimental terrains: flat ground with obstacles, 12° slope, flat grass field, and grass field with obstacles. The robot autonomously moved along a program-predefined route without prior information about the environment using the motion model described in Section 4. The robot’s pitch angle was calculated by the IMU installed on the robot at 10 Hz.

#### 5.3.2 Experiments on flat terrain with obstacles

**FIGURE 10:**
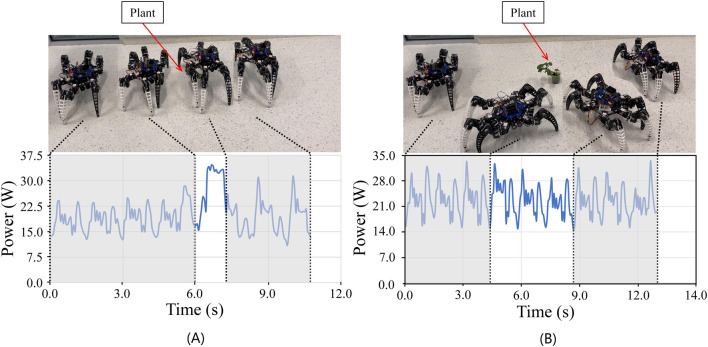
Two ways for a robot to cross an obstacle on a flat surface. **(A)**. The robot used the TA algorithm to lift the clearance to cross the obstacle. It took 10.7 s with an average CoT of 25.3. **(B)**. The robot used a conventional algorithm to avoid obstacles. It took 12.5 s with an average CoT of 30.2.

In order to evaluate our robots’ motion efficiency and energy consumption, we conducted controlled experiments. As shown in Figure 10, we divided the experiments into two groups. We set the robot at the same starting point, one robot executes the traditional tripod gait algorithm (Duan et al., 2009) to avoid obstacles following the path planned by the artificial potential field algorithm (Khatib, 1985), and the other executes our TA gait. The disadvantage of the traditional algorithm is that it can only go around obstacles and cannot cross them from above. We would like to emphasize that our robot can cross obstacles by lifting the clearance, thus saving time and energy. The final results demonstrate that the robot in the experimental group executing the TA gait completes avoiding obstacles and reaching the endpoint in only 10.7 s, which is 1.8 s (14.4%) less than the robot in the control group executing the traditional obstacle avoidance algorithm. Furthermore, voltage and current variations were directly recorded during the task, and the average CoT was calculated. The results demonstrated that the average CoT of robots executing the TA gait was 25.3, which was 16.2% lower than that of the traditional algorithm, which was 30.2 when obstacles of comparable size were avoided.

#### 5.3.3 Experiments on a slope

**FIGURE 11:**
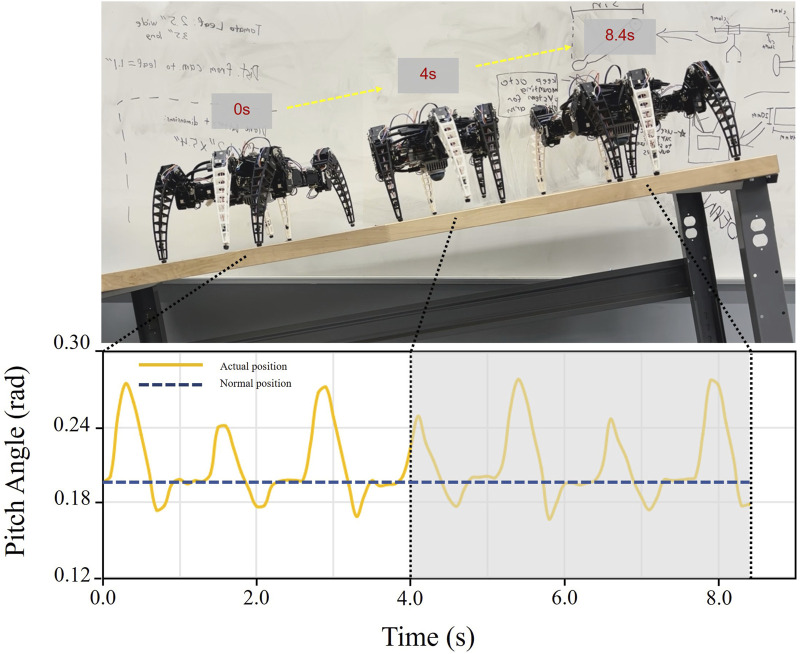
Variation in robot’s pitch angle during slope ascent, fluctuating between −2.3° and 4.6°, indicating stable attitude throughout the climb.

Figure 11 shows the corresponding pitch angle variation for the robot traveling on a 12° slope. The yellow line represents the average pitch angle changes for the 10 runs. Upon subtracting the slope gradient, the average pitch angle variation ranges from −0.04 to 0.08 radians or −2.3° to 4.6°. The experimental results demonstrate that our robot can maintain stable walking on a slope precisely.

#### 5.3.4 Outdoor experiments on a grass field

**FIGURE 12:**
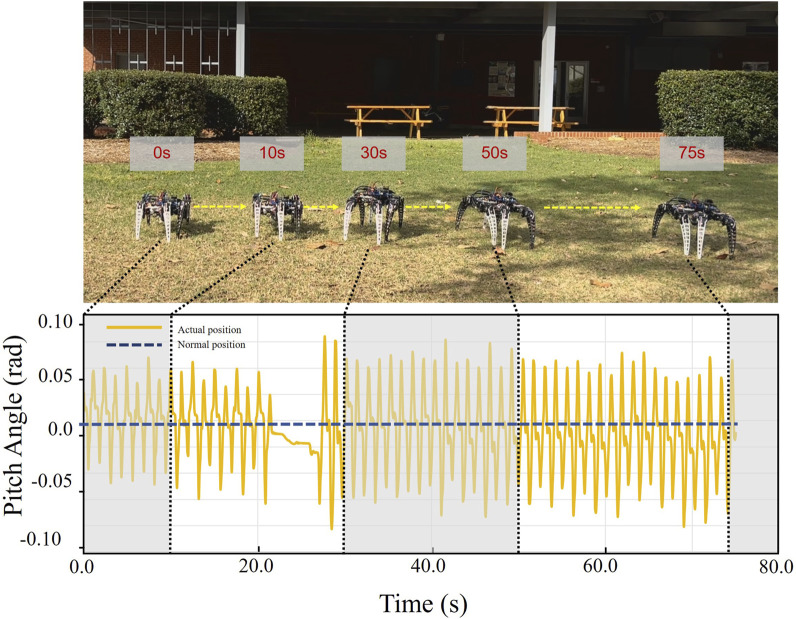
Variation in pitch angle as the robot navigates grassy terrain, ranging from −2.9° to 4.6°. This demonstrates the robot’s ability to maintain stability on deformable ground.

To test the robot’s ability to walk on deformable surfaces, we chose a grass field for the test. Figure 12 shows the corresponding pitch angle variation for the robot walking on the grass. The yellow line represents the average pitch angle changes for the 10 runs. It can be seen that the average pitch angle change ranges from −0.05 to 0.08 radians or −2.9° to 4.6°. The experimental results show that our robot can maintain stable walking on grass with consistent precision.

#### 5.3.5 Outdoor experiments on a complex grass field with obstacles

**FIGURE 13:**
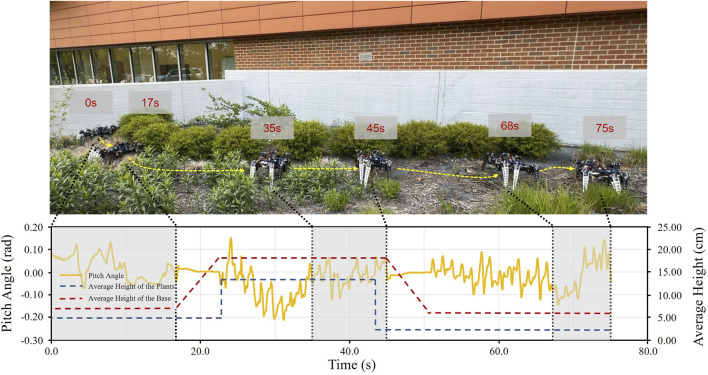
Variation of the pitch angle of the robot as it walks through a complex grassy field and crosses an obstacle. Under various ground conditions, the robot shakes considerably sometimes in the process, and the pitch angle varies from −11.5° to 8.6°. The results indicate that the robot can successfully pass through complex fields.

Based on the previous experiment, we added more complex variations, including plants of different heights, curved paths, and sandy and muddy fields with varying soil quality. This environment configuration can better simulate the environment in a real agricultural scenario. Figure 13 shows the corresponding pitch angle change for the robot traveling through this terrain. The yellow line represents the average of the pitch angle variation over 10 runs. It can be seen that the average pitch angle variation ranges from −0.20 to 0.15 radians or −11.5° to 8.6°. The pitch angle remains relatively constant when the robot raises and lowers the clearance. The experimental results show that our robot could maintain stable walking in the terrain smoothly switched clearance and avoided obstacles with impressive problem-solving skills.

## 6 Conclusion and future work

In summary, we have successfully developed a hexapod robotic system that demonstrates locomotion stability under various environmental conditions. The system has exhibited exceptional performance in both simulated environments and field tests. The main contribution of this robotic system is that it solves the problem that traditional agricultural robots are unable to traverse farmland with a cluttered ground environment. In such environments, crops are highly susceptible to damage. The highlight of our robot is that it executes terrain-adaptive gait algorithms that can switch motion modes according to different terrains, which enables it to cope with highly complex environments. Additionally, it has adaptive clearance, which allows it to directly cross obstacles without going around them. We have successfully modeled the robot’s movement over various terrains in simulation. In real-world tests, the robot performed well on slopes with a gradient up to 17°. The fluctuation of the center of gravity when crossing the obstacles was controlled from −2 to 2 cm. Finally, we also completed a large number of field tests. On the flat surface, our terrain-adaptive algorithm is more energy efficient than traditional obstacle avoidance algorithms, saving 14.4% of energy consumption for every obstacle crossed. In the tests on grassland, our robot still maintains good stability, with pitch angle fluctuations of only −11.5° to 8.6°. We provide the mean, standard deviation, and root-mean-square error of the pitch angle of the robot while moving over these three terrains in Table 2. The robot demonstrates remarkable stability in performance when navigating slopes and grass fields. Specifically, the root means square error (RMSE) of the robot’s pitch angle is 1.803° on slopes and 3.559° on grass fields. Notably, even under challenging conditions, such as traversing a complex grass field with uneven terrain and multiple obstacles, the RMSE of the robot’s pitch angle remains within an acceptable margin of error at 7.140°. Compared to previous work (Ouyang et al., 2021), our robot can walk stably on slopes with greater angles and with less oscillation on flat and complex terrain. However, there are still some practical problems that need to be solved. Our subsequent research will focus on the following three areas.

1. Improve stability: The robot’s mechanical structure and motion model will be enhanced. For instance, the leg degrees of freedom will be augmented to ensure that the foot end is vertically in contact with the ground. Furthermore, artificial intelligence methodologies can be employed to refine the motion model.
2. Improve endurance: To extend the operational time of the robot, it is necessary to optimize its energy consumption. This can be achieved not only by increasing the battery capacity and reducing the overall weight of the robot, but also by optimizing it using bio-inspired and deep learning approaches (Polykretis et al., 2020; Lopez-Osorio et al., 2022; Ehrlich and Tsur, 2021; Thor and Manoonpong, 2021). By utilizing these advanced techniques, the endurance of robots can be significantly improved.
3. Better intelligence: Further experiments are needed to develop the robot’s performance in more complex environments. We will deploy the robot in a real environment in a farm field to test its autonomous navigation and obstacle avoidance performance.

**TABLE 2:** Mean, standard deviation, and root mean square error of pitch angle for robot moving on 12° slopes, grass field, and complex grass field with obstacles.

Type of terrain	Mean/°	Standard Deviation/°	Root mean square Error/°
12° Slope	12.261	1.752	1.803
Grass Field	−1.417	3.267	3.559
Complex grass field with obstacles	1.580	6.968	7.140

## Data Availability

The raw data supporting the conclusions of this article will be made available by the authors, without undue reservation.
