# Supplementary material for: Development of a bionic hexapod robot with adaptive gait and clearance for enhanced agricultural field scouting
Source: Front Robot AI. 2024 Sep 18;11:1426269. doi: 10.3389/frobt.2024.1426269 (PMC11444934; doi:10.3389/frobt.2024.1426269)
Supplement: Supplementary file 1 [file DataSheet1.docx]

Supplementary Material

## Supplementary Figures


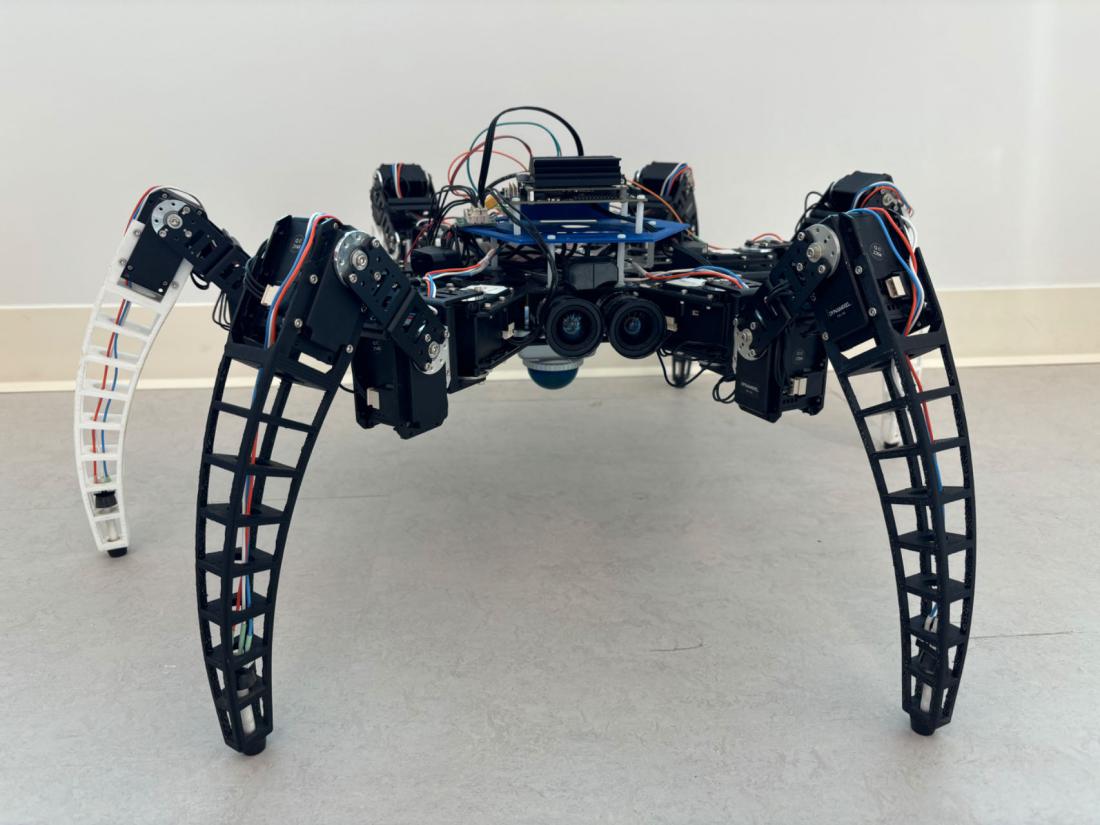


**Supplementary Figure 1.** The robot prototype


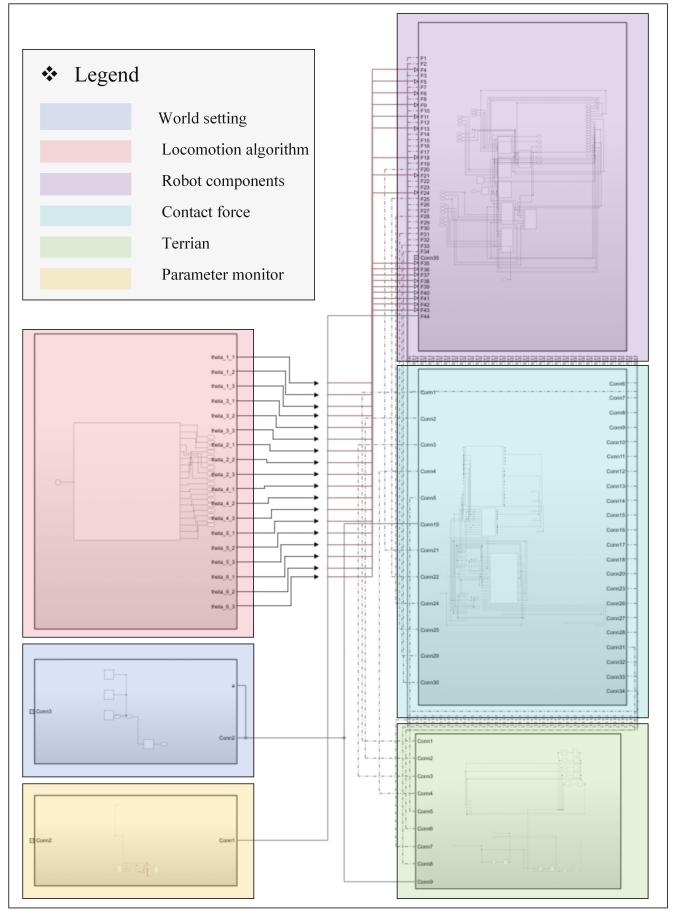


**Supplementary Figure 2.** Schematic overview of the simulation environment model of the robot system. The model includes robot components, environment parameters, motion algorithms, physical information, and parameter monitors.


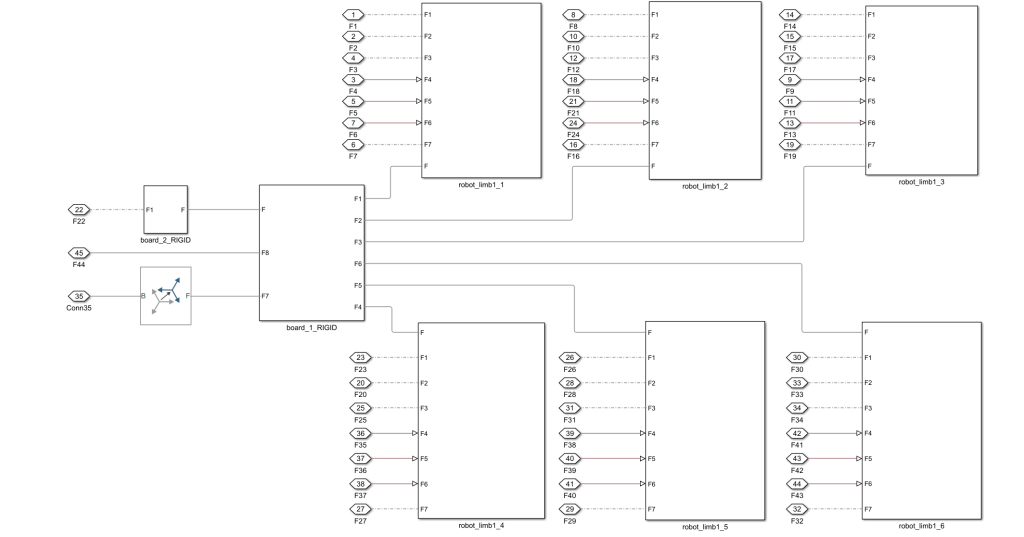


**Supplementary Figure 3.** Simulation model of the robot components


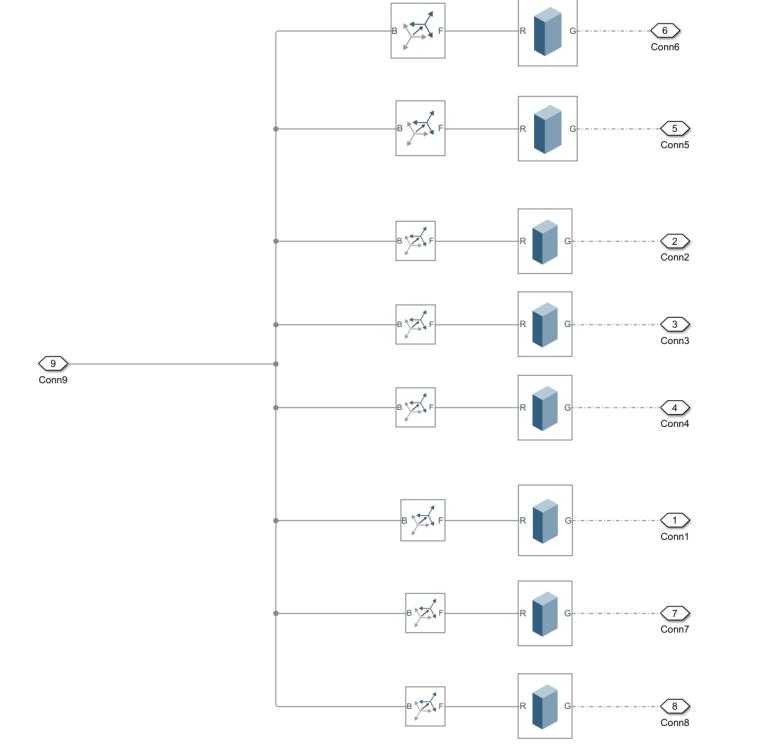


**Supplementary Figure 4.** Simulation model of the environment


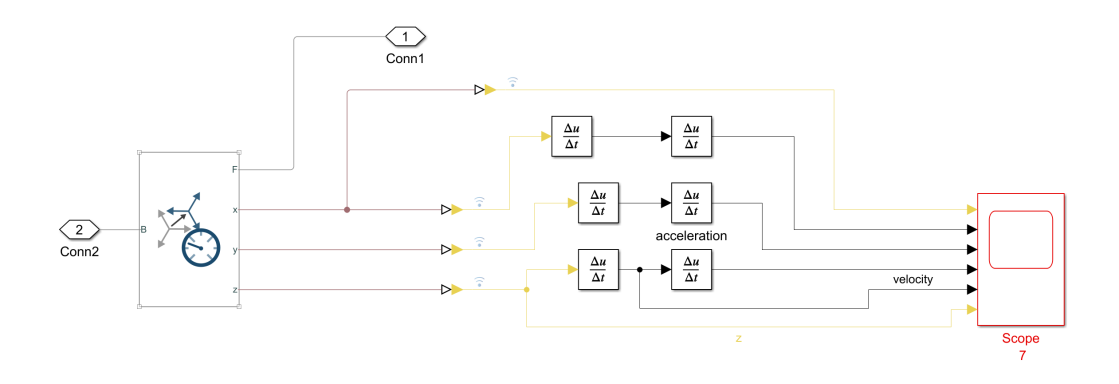


**Supplementary Figure 5.** Simulation model of the Parameter Monitor


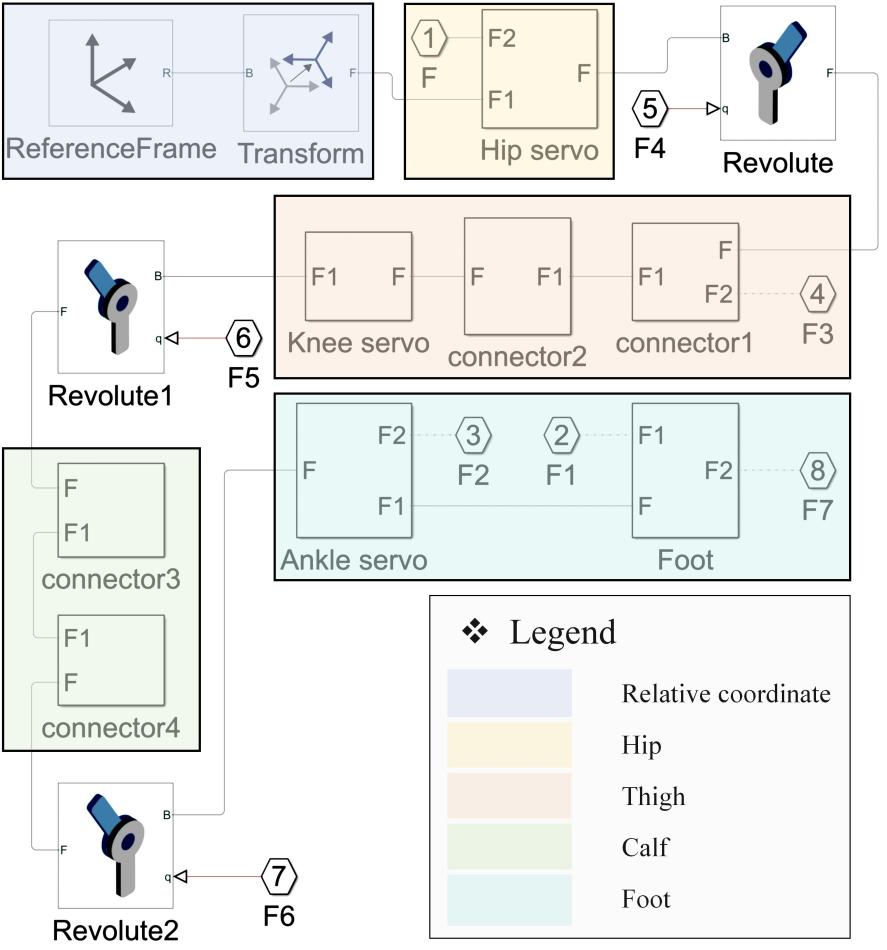


**Supplementary Figure 6.** Simulation model of the robot leg

**Supplementary Video 1.** https://drive.google.com/file/d/1FxqwH1kWIrcyO1V9pUpw3uP1AAjmXtGN/view?usp=sharing
